# Experimental Study on Active Interface Debonding Detection for Rectangular Concrete-Filled Steel Tubes with Surface Wave Measurement

**DOI:** 10.3390/s19153248

**Published:** 2019-07-24

**Authors:** Bin Xu, Lele Luan, Hongbing Chen, Jiang Wang, Wenting Zheng

**Affiliations:** 1 College of Civil Engineering, Huaqiao University, Xiamen 361021, China; 2 Key Laboratory for Intelligent Infrastructure and Monitoring of Fujian Province (Huaqiao University), Xiamen 361021, China; 3 Department of Civil and Environmental Engineering, Northeastern University, Boston, MA 02115, USA; 4 Department of Civil Engineering, Tsinghua University, Beijing 100084, China

**Keywords:** rectangular concrete-filled steel tube (RCFST), piezoelectric lead zirconate titanate (PZT), surface waves, interface debonding defect, defect detection

## Abstract

Concrete-filled steel tube (CFST) members have been widely employed as major structural members carrying axial or vertical loads and the interface bond condition between steel tube and concrete core plays key roles in ensuring the confinement effect of steel tube on concrete core. An effective interface debonding defect detection approach for CFSTs is critical. In this paper, an active interface debonding detection approach using surface wave measurement with a piezoelectric lead zirconate titanate (PZT) patch as sensor mounted on the outer surface of the CFST member excited with a PZT actuator mounted on the identical surface is proposed in order to avoid embedding PZT-based smart aggregates (SAs) in concrete core. In order to validate the feasibility of the proposed approach and to investigate the effect of interface debonding defect on the surface wave measurement, two rectangular CFST specimens with different degrees of interface debonding defects on three internal surfaces are designed and experimentally studied. Surface stress waves excited by the PZT actuator and propagating along the steel tube of the specimens are measured by the PZT sensors with a pitch and catch pattern. Results show that the surface-mounted PZT sensor measurement is sensitive to the existence of interface debonding defect and the interface debonding defect leads to the increase in the voltage amplitude of surface wave measurement. A damage index defined with the surface wave measurement has a linear relationship with the heights of the interface debonding defects.

## 1. Introduction

With advanced structural performance including high load-carrying capacity, good ductility and energy dissipation capability under strong dynamic excitations, convenience and economy in construction, concrete-filled steel tubes (CFSTs) have been extensively employed as major vertical and/or axial load-carrying structural members in civil infrastructure such as long-span bridges, super high-rise buildings and off-shore platforms in harsh environments [1]. Moreover, large-scale and complex CFST members with irregular cross section have been employed in practice to meet increasing load-carrying capacity and the accelerated construction speed needs [2]. The perfect confinement effect of steel tube on concrete core plays key roles in ensuring advanced mechanical properties of CFSTs. The compressive strength of concrete core increases due to the confinement effect from steel tube and the local buckling of steel tube as a thin wall structure can be avoided due to the existence of concrete core. Therefore, the interface bond condition between the steel tube and concrete core is a significant factor affecting the confinement effect and the performance of CFSTs. In practice, interface debonding defects may exist between the steel tube and the concrete core due to complex inner structure such as the horizontal diaphragms, obvious heat of hydration of mass concrete core, poor quality control for concrete pouring and so on. Xue et al. investigated the effect of interface debonding on the mechanical behavior of circular CFST columns subjected to axial load and eccentric load with results indicating that the interface debonding leads to serious local buckling of steel tube and decrease in the ultimate load-carrying capacity when compared with that of specimens without interface debonding defects [3].

In the last decades, various global structural identification approaches for different types of civil engineering structures have been investigated using structural dynamic characteristics such as frequencies, damping ratios and modal shapes extracted from structural dynamic response measurements [4,5,6]. Unfortunately, it is difficult to detect small local defects such as early-stage cracks or debonding defects in engineering structures by global dynamic parameters because the lower order modal shapes or frequencies are insensitive to local defects in practice. Therefore, novel local defects detection and non-destructive (NDT) approaches for civil engineering structures such as ultrasonic method, impact-echo method, vision method, and ray method have been developed [7,8,9,10]. However, most of the existing methods are ineffective for the interface debonding detection in CFST members. For example, although the microwaves method was proposed to detect interface damage of fiber reinforced polymer (FRP)-wrapped concrete structures [11], it is not suitable for CFST structures due to the shielding effect of steel tube, which makes the penetration of electromagnetic wave through the metallic media impossible. 

In recent years, piezoelectric lead zirconate titanate (PZT)-based approaches have been widely recognized as one of the most promising active structural health monitoring (SHM) techniques for engineering structures using stress wave measurement and electromechanical impedance [12,13,14,15,16]. The feasibility of PZT based defect detection approaches for L-shaped CFST columns, timber materials, prototype RC bridge, fiber-reinforced composites, steel-reinforced concrete (SRC), concrete structures repaired/reinforced with composite materials, and steel bridge components has been investigated experimentally and numerically [17,18,19,20,21,22]. In order to employ PZT patches in the SHM of concrete structures, a piezoceramic-based device known as smart aggregate (SA) has been proposed. Song et al. summarized its application in early-age concrete strength monitoring, impact detection and crack initiation and propagation of RC structures [23,24]. SAs were also used to detect the damage of circular RC columns and beams with wave measurement [25].

PZT based stress wave measurement approaches also play active roles in the bonding condition monitoring between concrete and rebar in RC structures [26,27]. Sharma and Mukherjee used guided waves propagating along steel rebars in concrete to evaluate the corrosion of rebar in varying environments [28]. Qin et al. developed the active sensing approach using SAs to detect the initiation and the development of bond-slip in two SC beams [29]. Zeng et al. used shear waves to monitor the bond slip in concrete-encased composite structures with embedded shear mode SAs [30]. De Marchi et al. proposed a time-frequency signal processing procedure aimed at extending pulse-echo defect detection methods [31]. Miniaci et al. proposed an ultrasensitive technique to detect and localize sources of elastic nonlinearity using phononic crystals and experimentally validated the approach [32]. Results show its potential as an efficient, compact, portable, passive apparatus for nonlinear elastic wave sensing and damage detection.

For the interface debonding detection of CFSTs, Xu et al. firstly proposed a novel PZT based active approach, where PZT patches mounted on the outer surface of the steel tube or embedded in concrete core are used as actuator or sensors and the changes in the wavelet energy and wavelet energy spectrum of the PZT sensor measurements are employed to detect the interface debonding defects, and the feasibility of the proposed approaches was validated experimentally and numerically considering the piezoelectric effect of PZT materials and the coupling effect between PZT patches and CFST members [33,34,35,36,37]. Most recently, for the purpose of investigating the effect of randomness of mesoscale structure of concrete core on the applicability of interface debonding detection approach, Xu et al. studied the dominance of debonding defect on PZT sensor response considering the mesoscale structure of concrete with multi-scale simulation [38]. In order to improve the computation efficiency, a Legendre polynomial based spectral element method (SEM) was developed to simulate the static and dynamic behavior of piezoelectric bimorphs and the wave propagation in the cross-section of CFST members [39,40,41]. 

In fact, in the above interface debonding detection approaches, the embedded PZT sensor measures the bulk wave traveling across the steel tube and concrete core. The shortages of the defect detection approaches using bulk wave include the wave attenuation in concrete core and the inconvenience of the installation of embedded SAs in concrete core before concrete pouring. The wave attenuation in steel tubes of CFST members is smaller than that in concrete and it is more attractive to develop interface debonding approaches using surface wave measurement for CFST members [42]. Schaal et al. provided an approach for crack detection in near surface of a thick aluminum plate based on wave conversion at delamination-like cracks [43]. Song et al. investigated the effect of microstructures on the surface wave propagation and showed the feasibility of using surface waves generated and received by PZT actuators/sensors for quantitative damage detection in concrete structures [44]. Park et al. presented a new impact localization technique that can pinpoint the location of an impact event within a complex structure using a time-reversal concept, surface-mounted piezoelectric transducers, and a scanning laser Doppler vibrometer [45]. Miniaci et al. employed a laser-based time-reversal algorithm for impact localization in a stiffened aluminum plate to cope with the material inhomogeneity or geometrical irregularities of the tested parts [46]. 

Most recently, a non-destructive early corrosion detection technique in steel tubes of CFST members using surface wave measurement was proposed and experimentally investigated [47]. However, the proposed approach needs a water tank to hold water on the surface of the steel tube as coupling media and a pair of transducers with identical inclination with the steel tube should be employed, which limits the application of this method in practice for large-scale CFST members as vertical load-carrying components. Moreover, the proposed guided Lamb wave measurement based notch and debonding detection approach are effective when CFST member can be recognized as a slender member. However, CFST members employed in high-rise building usually have a large cross-section and cannot be recognized as slender members. Therefore, it is desired to develop an active interface debonding detection approach using surface wave measurement by PZT patches mounted on steel tube surface of CFST excited by PZT actuators mounted on the identical surface of steel tube. Chen et al., proposed a transient multichannel analysis of surface waves (MASW) approach to detect the existence, the location and the length of interface debonding defects in rectangular CFST and carried out mesoscale numerical analysis to validate numerically the feasibility of MASW-based interfacial debonding detection approach [48]. Further experimental investigation is desired to test the feasibility of the surface wave based interface debonding detection approach. 

In this study, to overcome the shortcomings of the current interface debonding detection approaches using embedded PZT patches, an active interface debonding detection approach using surface wave measurement with PZT patches mounted on the identical surface of steel tube of CFST member as actuator and sensors is proposed. In order to demonstrate the feasibility and the performance of the proposed approach, experimental studies on two rectangular CFST members with different interface debonding defect scenarios are carried out. The measurements of the surface waves propagating from the surface-mounted PZT actuator and passing through different interface debonding defects are compared. The relationship between the measurement and the interface debonding widths and heights is investigated. An evaluation index is defined based on the surface wave measurement and its linear relationship with the heights of the interface debonding defects is found. Experimental results show the proposed approach is efficient for interface debonding condition monitoring for CFST members with surface wave measurement.

## 2. Interface Debonding Detection Approach with Surface Wave Measurement

**Figure 1:**
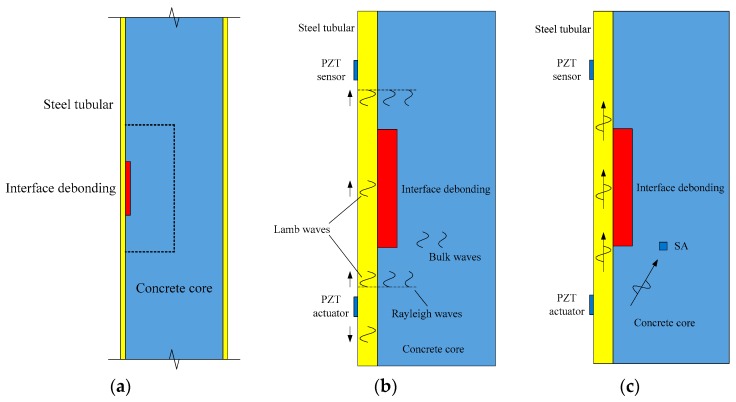
Concept of debonding detection using surface wave measurement for a concrete-filled steel tube (CFST) member. (**a**) CFSTs with interface debonding; (**b**) wave propagation in CFST; (**c**) arrangement of piezoelectric lead zirconate titanate (PZT) patches.

The interface debonding defect approach presented in this study uses surface wave measurement along the steel tube. Figure 1a shows a longitudinal section of a CFST member with an interface debonding. As shown in Figure 1b, in order to detect the interface debonding defect, both PZT actuator and sensor are bonded on an identical outside surface of the steel plate. Under the excitation of the input voltage signal, the PZT actuator produces stress wave propagating in steel plate as well as core concrete, which includes bulk waves (P and S waves) and Rayleigh waves [43]. Except for the bulk waves and Raleigh waves, the excitation of PZT actuator also generates Lamb waves in the steel tube when interface debonding defect occurs as shown in Figure 1b. The conversion of Rayleigh to Lamb wave at the debonding defect leads to the changes in waveform and wave energy, which can be measured by PZT sensors [43]. Therefore, it is reasonable to detect the existence of interface debonding defect from the variation in the surface wave measurement from PZT sensor. Moreover, in order to investigate the change in bulk wave progatating from the PZT actuator and across the interface debonding defect, SAs are embedded in the concrete core as shown in Figure 1c.

## 3. Experimental Setup and Interface Debonding Detection with Surface Wave Measurement

### 3.1. CFST Specimens with Interface Debonding Defects

**Figure 2:**
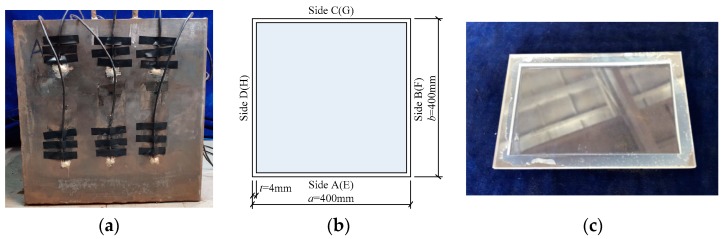
CFST specimen with surface-mounted PZT patches and its cross-sectional dimension (unit: mm). (**a**) Surface-mounted PZT patches; (**b**) cross-sectional dimension; (**c**) an acrylic plate.

In this study, two rectangular CFST specimens with identical dimensions were designed. Figure 2a shows a rectangular CFST specimen, where a number of PZT patches acting as either actuators or sensors are bonded on its outer surface. Each CFST column specimen studied here had an outer cross-section of *a* × *b* = 400 mm × 400 mm and a height of 400 mm, as shown in Figure 2b. The thickness of the steel tube *t* was 4.0 mm. The four sides of the specimen No. 1 were named Sides A, B, C and D, while those of the specimen No. 2 were labeled as Sides E, F, G and H, as illustrated in Figure 2b. For comparison, three sides of each specimen had interface debonding mimicked by pasting soft materials on the inner surface before pouring concrete and one side had no debonding defect. Figure 2c shows an acrylic plate with a groove to be pasted on the inner surface of the steel tube before pouring concrete. The thickness of the center groove part of the acrylic plate was 1.5 mm and the thickness of the frame along the boundaries was 3.5 mm. When the acrylic plate was pasted on the inner surface of the steel tube, a gap between steel tube and concrete core with a thickness of 2 mm was mimicked.

**Figure 3:**
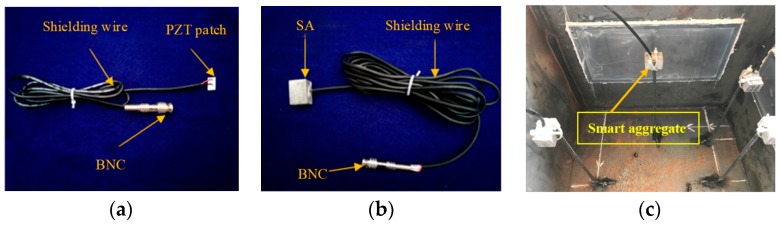
Sample of PZT sensors and smart aggregate (SA) installation. (**a**) Surface bonded PZT patch; (**b**) embedded SA; (**c**) establishment of SAs.

As shown in Figure 3a, the employed PZT patches with dimensions of 15 mm × 10 mm × 0.3 mm were polarized along their thickness direction. The detailed material parameters of the PZT patch were identical to that of the numerical research performed by Xu et al. [35,36,37,38,39,40]. Figure 3b shows an SA to be embedded in concrete core, which was used for a comparative study and was not necessary when using the proposed debonding detection approach using surface wave measurement, which only requires surface-mounted PZT patches. The detailed description of the preparation and fabrication of the surface-mounted PZT patches and SAs has been made in previous studies [33,34]. In addition, corresponding to each side of the specimen, an embedded SA was used to measure the wave propagating from the steel tube into the concrete core. Figure 3c shows the installation of SAs in a CFST specimen before casting concrete, where the SA was fixed on a thin steel bar apart from the inner surface of the steel tube.

In this study, six interface debonding scenarios were designed and the corresponding dimensions of each mimicked debonding defect are shown in Table 1. It should be noted that the sizes of debonding defects were determined by the outer contour dimensions of the rigid acrylic plates.

### 3.2. Surface Mounted PZT Actuators, Sensors and Embedded SAs

**Figure 4:**
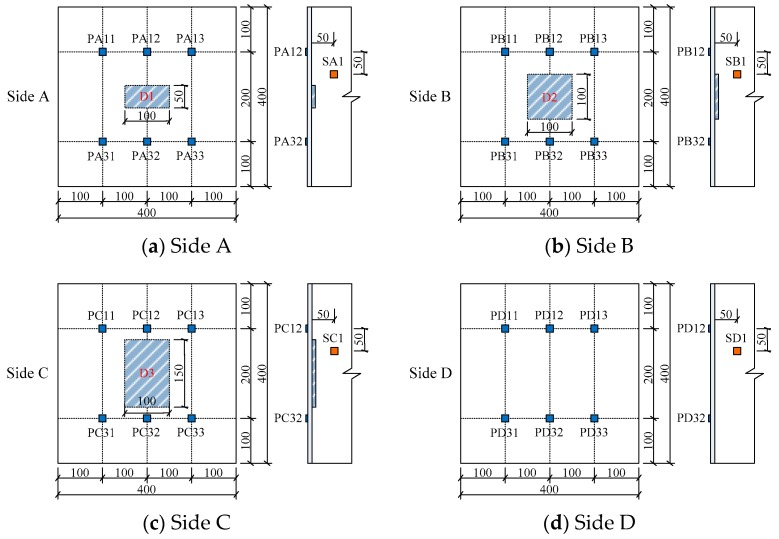
Arrangement of PZT patches, SAs and debonding defects in specimen No. 1 (unit: mm).

**Figure 5:**
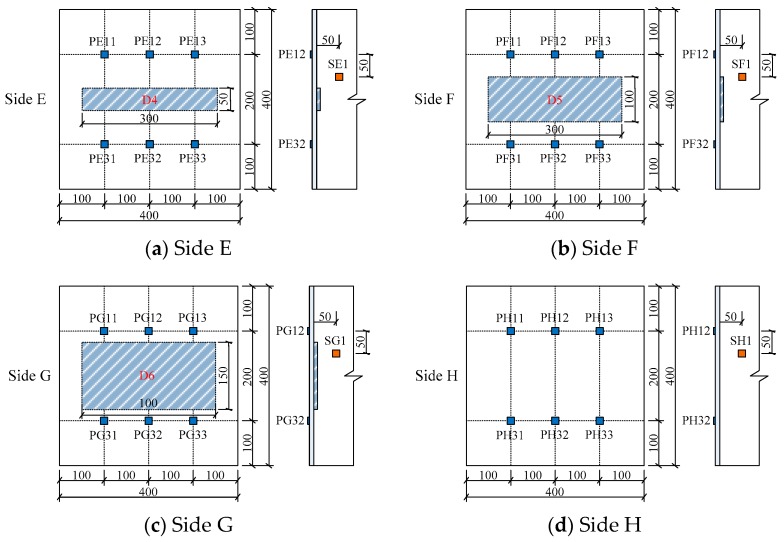
Arrangement of PZT patches, SAs and debonding defects in specimen No. 2 (unit: mm).

The arrangement of PZT actuators and sensors mounted on the identical outer surfaces of the two tested specimens, the embedded SAs and the location of the mimicked interface debonding defects shown as shaded regions are illustrated in detail in Figure 4 and Figure 5, respectively. The surface-mounted PZT patches on each side were distributed with a 2 × 3 array. For each side, the PZT patches in upper row were used as actuators while the PZT patches in lower row were used as sensors. For example, the surface mounted PZT patches on Side A, PA11-PA31, PA12-PA32, and PA13-PA33, formed three pitch and catch patterns. 

As shown in Figure 5a, the SA1 was employed to measure the stress wave in the concrete core due to the excitation of the PZT actuator labeled as PA12.

### 3.3. Test Setup of Surface Wave Measurement Based Interface Debonding Detection with PZT Patches

A continuous sinusoidal signal with a frequency of 10 kHz and an amplitude of 10 V generated by an arbitrary waveform/function generator was used to excite the PZT actuators on each side of the tested specimen. The output voltage signals of both PZT sensor and the embedded SA sensor were recorded using a high-frequency data acquisition system with a sampling frequency of 102.4 kHz. 

## 4. Experimental Results Analysis for Interface Debonding Detection

**Figure 6:**
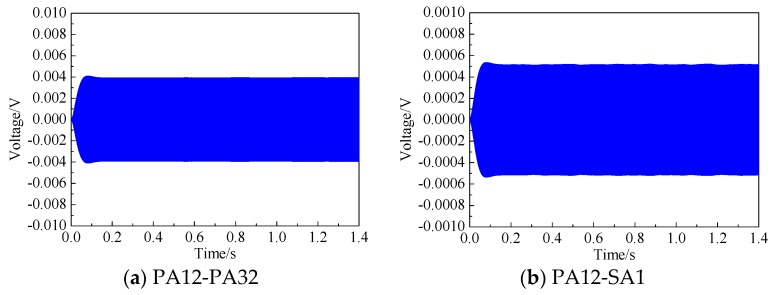
Voltage response of PA32 and SA1 under the excitation of PA12 on Side A.

Figure 6 presents the voltage signals measured by surface-mounted PZT sensors of PA32 and SA1 under the excitation of PA12 on Side A after filtering. In the following study, the voltage signals measured by all sensors were filtered and the signals from 0.5 s to 0.501 s with a time duration 0.001 s were selected for comparison. Here, the voltage responses measured by the surface-mounted PZT sensors on two tested CFST columns were employed to analyze the effect of interface debonding detects on the waves propagating along the specimen surface and into the concrete core.

### 4.1. Effect of Interface Debonding on Surface and Bulk Wave Propagation

**Figure 7:**
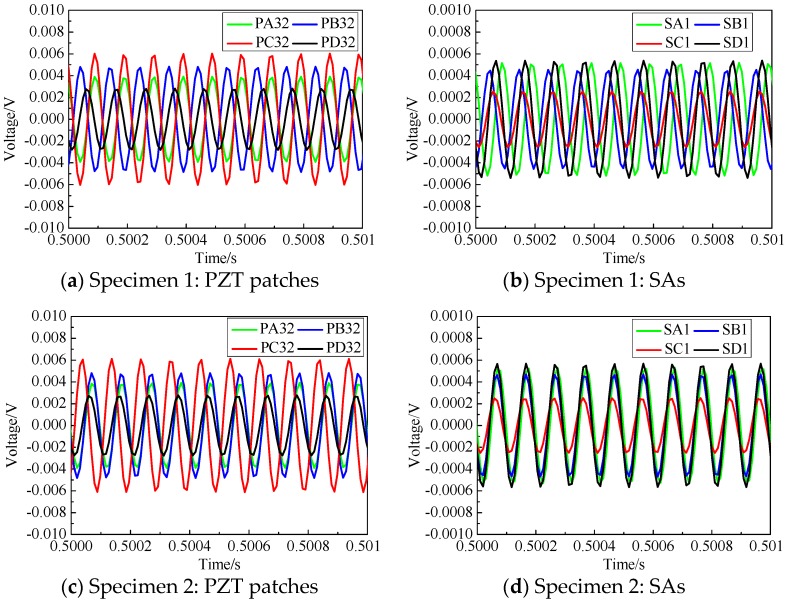
Voltages measurement of selected PZT patches and SAs in each side of tested specimens.

Figure 7 shows the comparison of the response voltages measured by PZT sensors and SAs on each side of the two CFST specimens during the selected time duration. It can be seen that the PZT sensor measurement on Side D without interface debonding was the smallest and the PZT sensor measurement increased obviously when the areas of the interface debonding defects increased. The amplitude of the PZT sensor measurement on Side C with the largest interface debonding defect was the highest. The amplitude of the measurement of the SAs showed an inverse trend. The amplitude of the SA measurement corresponding to Side D was the largest and that corresponding to Side C was the smallest. The PZT patches measured the waves propagating along the surface of the CFST specimen including Lamb waves and Raleigh waves, while the SAs measured the bulk waves propagating into concrete core from the PZT actuator. The difference in the measurement amplitude by PZT sensor measuring the surface wave propagation along the steel tube makes it possible to identify the existence of interface debonding defect. 

The interface debonding defect may attenuate or block the stress wave propagation from steel tube to concrete core, and then lead to the decrease in the measurement of SA sensors and the increase in the amplitudes of the voltage signals measured by PZT patches. Moreover, from the test measurements, it can be seen that the amplitude of the response voltage induced by the surface waves increased with the enlargement of the debonding height. The reduction of the voltage amplitude measured by SAs due to the existence of debonding defect met the findings from the collected literature [33,34].

### 4.2. Interface Debonding Detection

To further demonstrate the efficiency of the proposed surface wave measurement based interface debonding detection approach, the measurement from three PZT sensors on an identical side of each specimen were compared and analyzed. 

**Figure 8:**
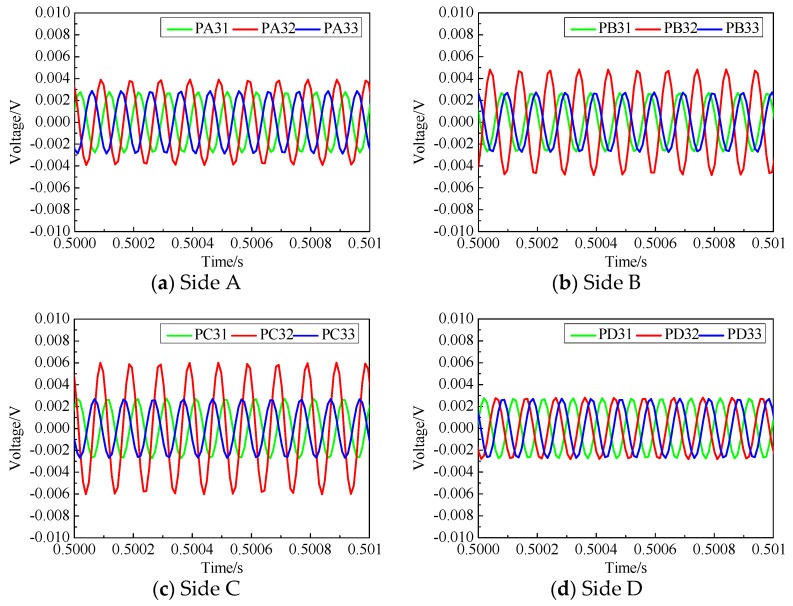
Voltage measurement of the surface-mounted PZT sensors in specimen No. 1.

**Figure 9:**
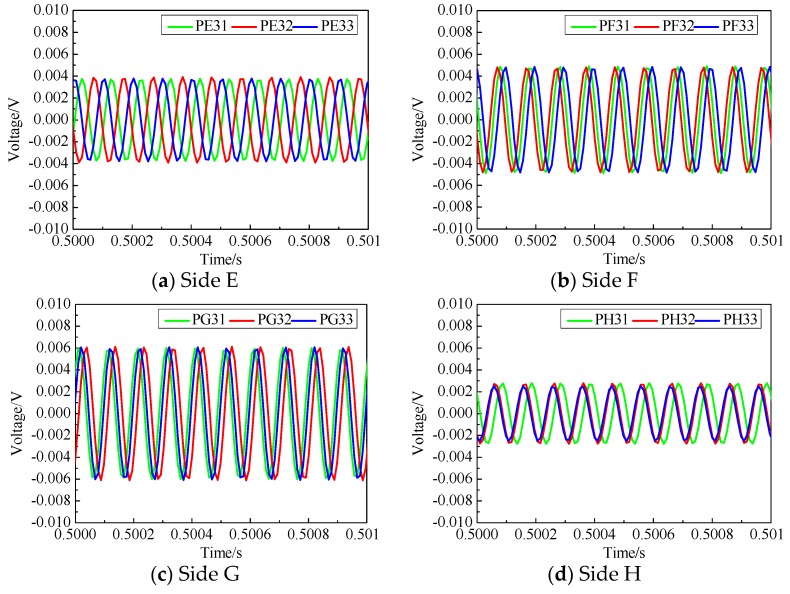
Voltage measurement of the surface-mounted PZT sensors in specimen No. 2.

Figure 8 and Figure 9 are the voltage measurements obtained from the PZT sensors on each side of these two tested CFST specimens. As shown in Figure 4, the width of each interface debonding defect in specimen No. 1 were identical, but the height of each interface debonding defect was different. Also, the interface debonding defects on Sides A, B and C were located in the travel path of the surface wave in the second pitch and catch pattern but were out of the connection lines of the first and the third pitch and catch PZT patches. As shown in Figure 8d, the measurement amplitudes of the three surface-mounted PZT sensors on Side D with no debonding defect were very close. Figure 8a–c show the measurement results of three PZT sensors on each side of Specimen No. 1 and it is clear that the amplitude of the voltage signal of the PZT sensor in the second column was larger than that of the two sensors on the left and right columns where no interface debonding defect exists along the surface wave propagation path. When there is no interface debonding defect between the actuator–sensor pairs, the voltage amplitudes of two different sensors were very close. Comparing the measurement of the PZT sensors of PA32, PB32 and PC32 shown in Figure 8a–c, it can be found that the measured voltage amplitudes increased obviously with the increment of debonding defect heights.

As detailed in Figure 5, the width of each interface debonding defect of the tested CFST specimen No. 2 wass identical but the height of each interface debonding defect was different. Unlike the specimen No. 1, the width of the interface debonding defects covered the three traveling paths corresponding to three actuator and sensor pairs. As shown in Figure 9, compared with the voltage signal of the PZT measurement on Side D without interface debonding, the amplitude of the measurement signals of all PZT patches on other three sides showed an obvious increase due to the existence of interface debonding defects. Moreover, the amplitudes of the measurements from all three PZT sensors were very close. The amplitudes also increased with the height of debonding defect, which means interface debonding defects blocked the wave propagation from the steel tube to the concrete core. These results coincide with the experimental observations of the CFST specimen No. 1.

### 4.3. Interface Debonding Damage Index and Its Sensitivity

Here, the voltage amplitude of the output signals from the PZT sensors is employed to identify the existence and to evaluate the degrees of interface debonding. As the width and thickness of interface debonding were constant for each specimen, the relationship between the variance of voltage amplitude with the height of the defects was investigated. A damage index (*DI*) based on the measured signal amplitude defined as follows was employed to reflect the degrees of interface debonding defects.

(1) $$DI\left(n\right)=\left|\frac{Dn-H}{H}\right|\times 100\%$$

where *Dn* and *H* respectively denote the amplitude of the voltage signals measured by PZT patch *n* located in the areas with and without interface debonding. 

**Figure 10:**
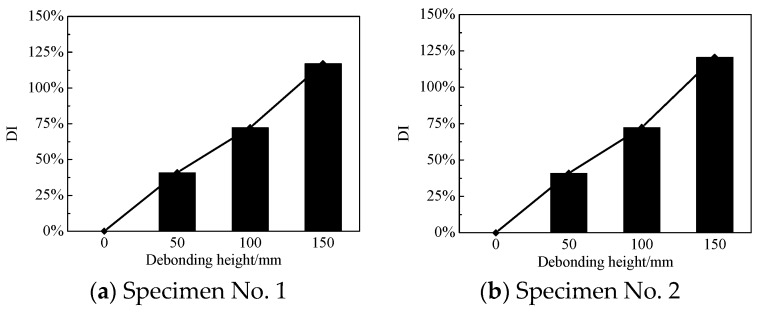
Damage index varying with the height of interface debonding.

Figure 10 shows the comparison of values of *DI(n)* corresponding to PZT sensors in the second column of each side of the two tested specimens, which changed with the height of interface debonding defects, respectively. It can be seen that this damage index was sensitive to the existence of debonding defect located in the surface stress wave propagation path from the actuator to the sensor. Moreover, the proposed *DI* had a clear linear relationship with the change in debonding height no matter what the width of the interface debonding defect was. 

**Figure 11:**
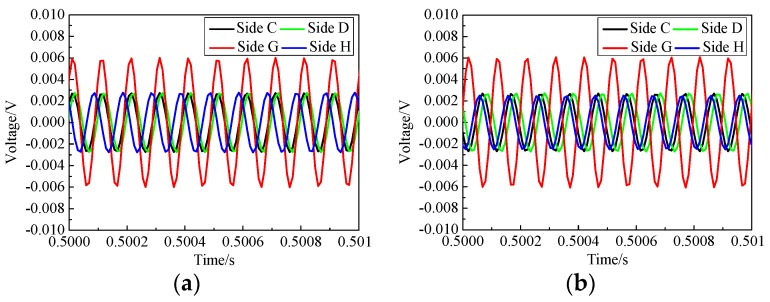
Voltage signals measured by the sensors in the first and third columns on Sides C, D, G and H. (**a**) Measurements on the first column; (**b**) measurements on the third column.

Based on the relationship between *DI* and the height of interface debonding, the height of interface debonding can be identified quantitatively, which is very meaningful in practice. Here, the measurements of the PZT sensors on the first and the third columns on Sides C, D, G and H are compared. As shown in Figure 4 and Figure 5, the heights of the two interface debonding on Sides C and G were identical but the widths were different. The interface debonding on Side G was wider than the area covered by the actuators and sensors in the first and the third pitch and catch patterns. Sides D and H had no interface debonding defects. Figure 11a shows the comparison of the sensor measurements on the first pitch and catch pattern on the four sides. It can be found that the amplitude of the sensor measurement on Side G was the highest and the amplitudes of the other three measurements were very close. It can be concluded that the interface debonding does not pose obvious effects on the measurement of the PZT sensors in the first pitch and catch pattern on Side C since the interface debonding width was apart from the surface stress propagation path from the actuator to the sensor in the first pitch and catch pattern. As shown in Figure 11b, similar findings can be observed from the comparison of the PZT sensors in the third pitch and catch pattern on different sides.

It is clear that the interface debonding detection approach using surface stress wave measurement with a pitch and catch pattern is capable of detecting interface debonding defects along the surface stress wave propagation path. However, the proposed method is insensitive to the interface debonding defects apart from the propagation path of surface stress wave. In practice, scanning the surface with a smaller interval is helpful to detect the width of interface debonding if a pitch and catch measurement pattern is employed.

## 5. Concluding Remarks

This paper proposed a surface wave measurement based active interface debonding defect detection approach for CFST structures using PZT actuating and sensing technologies. This approach only uses the PZT patches mounted on the outside surface of the steel tubes and there is no need to embed PZT transducers in concrete core. To demonstrate the efficiency of the proposed method, two CFST specimens with different degrees of interface debonding defects are established for comparison. Besides the surface-mounted PZT patches, SAs embedded in concrete core are employed to investigate the effect of interface debonding on the wave propagation along the surface and into the concrete core of CFST excited by PZT actuators. A continuous sinusoidal signal is applied to excite PZT actuators and the response voltage signals measured by the surface-mounted PZT sensors are analyzed to demonstrate the efficiency of the proposed method in detecting the existence and degrees of debonding defects in CFST structures. Based on the experimental study, the following conclusions can be made:

1. Interface debonding leads to an increase in the voltage amplitude measured by surface-mounted PZT patches and a reduction in the voltage amplitude measured by embedded SAs. Interface debonding blocks the propagation of stress wave from steel tube to the concrete core. Therefore, the increase in the voltage amplitude can be used to evaluate the existence of interface debondings;
2. When the width of interface debonding is constant, the measured voltage amplitudes present an obvious increment with the enlargement of debonding defect heights. The amplitude of the voltage signal has a clear relationship with the change in debonding height. The defined index is efficient to identify the existence as well as the height of interface debonding;
3. The PZT sensor measurement is sensitive to the existence of interface debonding defect along the stress wave propagation path from the actuator and sensor on the surface of CFST structures with a pitch and catch measurement pattern.

The surface wave measurement based debonding detection approach is convenient when compared with the bulk wave measurement-based approach where embedded SAs are required. Further experimental studies on the feasibility of the proposed approach considering the randomness and irregularity of interface debonding defects and numerical studies on the wave propagation mechanism will be carried out for practical application. 

## Figures and Tables

**Table 1:** Geometric parameters of the artificially mimicked interface debonding defects.

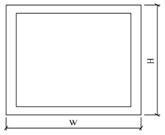	**Debonding Defect No.**	**Width (mm)**	**Height (mm)**
	D1	100	50
	D2	100	100
	D3	100	150
	D4	300	50
	D5	300	100
	D6	300	150
